# Impact of the Osijek Multidisciplinary Biopsychosocial Program on Chronic Low Back Pain: A Prospective Randomized Controlled Study

**DOI:** 10.3390/ijerph23030350

**Published:** 2026-03-11

**Authors:** Dijana Hnatešen, Ivan Radoš, Iva Dimitrijević, Dino Budrovac, Vanja Matković, Ivana Gusar

**Affiliations:** 1 Department of Pain Management, University Hospital Osijek, 31000 Osijek, Croatia; dijana.hnatesen@kbco.hr (D.H.); iva.dimitrijevic@kbco.hr (I.D.); dino.budrovac@mefos.hr (D.B.); vanja.matkovic@kbco.hr (V.M.); 2 Nursing Institute “Professor Radivoje Radić”, Faculty of Dental Medicine and Health Osijek, Josip Juraj Strossmayer University of Osijek, 31000 Osijek, Croatia; 3 Faculty of Medicine, Josip Juraj Strossmayer University of Osijek, 31000 Osijek, Croatia; 4 Department of Physiotherapy, College of Applied Sciences “Lavoslav Ružička”, 32000 Vukovar, Croatia; 5 Department of Health Studies, University of Zadar, 23000 Zadar, Croatia; igusar@unizd.hr

**Keywords:** low back pain, chronic pain, life quality, multidisciplinary treatment, multimodal treatment

## Abstract

This study aimed to assess the relative impact of the Osijek multidisciplinary biopsychosocial program for chronic low back pain (CLBP) compared with standard multimodal care with respect to pain intensity, disability, health-related quality of life, anxiety, depression, stress, and sleep quality using standardized self-assessment questionnaires and a smartwatch. A total of 128 patients treated at the Department of Pain Management, University Hospital Osijek, were randomly allocated to two groups. The multidisciplinary biopsychosocial group participated in a structured four-week program combining education, exercise, and individualized multidisciplinary care, while the multimodal group received conventional conservative treatment including pharmacotherapy and selected physical therapy modalities. The four-week intervention included standardized self-report questionnaires, a sociodemographic data form, and a Fitbit Charge 3 smartwatch for objective monitoring of sleep and physical activity. A significant reduction in pain intensity was observed across numerical scales and most questionnaire measures (Wilcoxon test, *p* < 0.01), except for the subscale assessing difficulties in performing daily activities due to sleep deprivation. Participants who underwent the multidisciplinary biopsychosocial treatment exhibited significant improvements (*p* < 0.03) in pain intensity, disability, health-related quality of life, stress, anxiety, and sleep quality compared with those receiving multimodal treatment. In both groups, a weak negative correlation was found between sleep quality and daily step count (Spearman’s rho = −0.234, *p* = 0.04). A multidisciplinary biopsychosocial program was associated with greater improvements in health-related quality of life, psychological well-being, and sleep quality in patients with chronic low back pain compared with a multimodal conservative approach. Increased daily physical activity was linked to improvements in anxiety and sleep. Although this study was designed as a randomized controlled trial, certain baseline differences between groups should be considered when interpreting the findings.

## 1. Introduction

Successful pain management is frequently described in the literature as a moral imperative [1,2] and as a fundamental human right [3,4]. Within this context, it becomes evident that the healthcare system and its stakeholders hold an ethical mandate to provide effective pain management and to uphold the dignity of every patient. Nevertheless, studies show that as many as 68% of individuals with chronic pain perceive their pain as inadequately managed [5], while other reports suggest that up to 80% of patients express dissatisfaction with their pain treatment [4]. In 2020, low back pain affected 619 million people worldwide, and the number of cases is projected to rise to 843 million by 2050, primarily due to population growth and aging [6]. The World Health Organization (WHO) defines health as “a state of complete physical, mental and social well-being and not only the absence of disease or infirmity” [7]. Meanwhile, according to its revised definition, the International Association for the Study of Pain (IASP) describes pain as an unpleasant sensory and emotional experience associated with actual or potential tissue damage, or resembling such an experience, emphasizing that pain is always influenced to varying degrees by biological, psychological, and social factors and is inherently shaped by personal experience [8]. It is important to note that these definitions underscore and advocate for a biopsychosocial approach to chronic pain management, given its multidimensional impact on all aspects of a patient’s life [9]. Taken together, these definitions are frequently cited as providing a conceptual basis for a biopsychosocial approach to chronic pain management [7,8]. However, their broad scope offers limited guidance on how this perspective should be consistently implemented in clinical practice. Since the Charter of Human Rights also defines the individual’s right to a life free from pain, which implies the necessity of ensuring adequate pain management, it is essential to give greater attention to chronic pain as a global health priority so that healthcare systems can fulfill their duty of providing accessible and standardized specialized care [10,11]. While the Charter of Human Rights frames freedom from pain as a fundamental right and implies the need for adequate pain management, the extent to which chronic pain is effectively prioritized as a global health concern remains unclear, particularly regarding the consistent provision of accessible and standardized specialized care within healthcare systems. In this context, the complexity of chronic pain and its multifaceted consequences further underscore the need for integrated and coherent management approaches.

Evidence indicates that patients who progress to the chronic phase of low back pain benefit from multidisciplinary approaches, and several recent guidelines advocate multidisciplinary biopsychosocial rehabilitation (MBR) for the management of chronic low back pain (CLBP) [12,13]. The effectiveness of multidisciplinary programs has been shown to surpass that of standard treatment and other non-multidisciplinary interventions [14]. According to the European Pain Federation (EFIC), multiple disciplines are not strictly required to implement a biopsychosocial approach to pain management. A single discipline may employ more than one strategy to address the biological, psychological, and social factors influencing a patient’s pain. For example, if one clinician integrates evidence-based pharmacotherapy with patient education, psychological approaches, and social support interventions, this can be regarded as a biopsychosocial approach. Exercise may likewise be applied to target specific biopsychosocial factors depending on treatment goals. However, a multidisciplinary or interdisciplinary approach (involving several disciplines) may be necessary for certain patients, both for pain management and for pain assessment. Treatments such as pharmacotherapy, interventional pain procedures, and manual therapy are commonly considered to target biological factors. Psychological approaches and patient education are viewed as targeting psychological factors, whereas workplace interventions or social support measures predominantly address social determinants of pain. What constitutes a biopsychosocial approach (including the number of disciplines or types of treatment needed) may vary by country, pain condition and duration, time frame and frequency of treatment sessions, and the availability of services capable of providing interdisciplinary care (e.g., physicians, physiotherapists, psychologists, nurses, and social workers collaborating as a team), all of which influence the formulation of an appropriate diagnosis and treatment plan [15]. Within contemporary pain management frameworks, a biopsychosocially oriented multidisciplinary approach represents the benchmark model for the treatment of chronic pain [16]. Quality of life is a broad concept encompassing all aspects of an individual’s existence, whereas health-related quality of life (HRQoL) focuses specifically on those components of quality of life that relate to an individual’s health [17], including levels of everyday functioning and the ability to experience a fulfilling life [18]. CLBP is one of the most prevalent chronic pain disorders, associated with a substantial burden for both individuals and society, and can significantly affect an individual’s HRQoL [19]. It is strongly linked to high pain intensity and disability, poorer prognosis, reduced HRQoL, marked physical limitations [20], and work incapacity [21]. Although the IASP provides a formal definition of multidisciplinary treatment as a multimodal approach [22], there remains considerable inconsistency in the use of related terminology. Terms such as multidisciplinary, interdisciplinary, and multimodal are frequently used interchangeably in the literature with varying interpretations and applications across rehabilitation programs. Despite the inconsistent terminology, the effectiveness of multidisciplinary and interdisciplinary teams in the treatment of chronic pain is widely documented, recognized, and accepted in health research [23,24,25,26]. Multidisciplinary and interdisciplinary treatment employing a biopsychosocial approach is considered the gold standard in the management of chronic pain [27,28], including CLBP. MBR program for the treatment of low back pain (LBP) for individuals with low back pain integrates physical therapy with psychological, social, and occupational components, aiming to restore pain- related functional limitations, recognize psychological factors, and address social and work-related challenges [29,30]. In addition, these programs seek to promote engagement in everyday activities and enhance overall quality of life [31]. According to the literature, multidisciplinary rehabilitation programs typically incorporate education on pain mechanisms and psychological aspects of pain, along with general health promotion, self-management strategies, goal-oriented planning, and structured exercise interventions. It should be emphasized that education should be delivered by all team members within the scope of their expertise, with the intent of helping patients reframe their understanding of pain into a less threatening one [31]. Specialized pain management also provides structured support by promoting the development of coping strategies, teaching pain management techniques, and addressing cognitive patterns such as pain catastrophizing, with the aim of reducing pain while shifting the therapeutic focus toward the restoration of functional daily living rather than the exclusive reduction pain intensity [32]. 

According to the IASP, multimodal treatment is defined as the simultaneous application of distinct therapeutic interventions with different mechanisms of action, delivered within a single discipline and targeting various pain mechanisms [22]. Multimodal pain management is provided by professionals from different disciplines, such as physicians, physiotherapists, and psychologists. Treatment typically includes a combination of exercise, patient education, relaxation techniques, and pharmacotherapy [33]. Although multidisciplinary and multimodal approaches may include overlapping therapeutic elements, the key distinction lies in their conceptual framework. Multidisciplinary treatment is defined by the involvement of professionals from different disciplines working collaboratively within a biopsychosocial model, whereas multimodal treatment refers to the concurrent use of different therapeutic modalities, which may be delivered within a single discipline and target different pain mechanisms [15,22]. In this study, the term “multimodal” refers to the combination of physical therapies, acupuncture, and pharmacotherapy, whereas “multidisciplinary” denotes the coordinated involvement of multiple health professionals delivering an integrated biopsychosocial intervention. The primary aim of a multidisciplinary program for chronic pain management is to enable the patient to assume an active role in managing their pain rather than remain a passive recipient of treatment. This goal is typically pursued through patient education, promotion of self-management strategies, active participation in therapeutic exercise and goal setting, and the development of coping skills that foster autonomy in pain management [32]. The multidisciplinary approach to chronic pain treatment has been present for decades as a therapeutic concept that has undergone numerous variations and transformations over time. Today, the multidisciplinary biopsychosocial approach is regarded as the gold standard in the management of chronic pain, including CLBP as the most prevalent pain condition globally, with all its associated individual, public health, and socioeconomic consequences.

Given the differences in the structure and implementation of multidisciplinary programs across clinical settings, the aim of this study was assessing the relative impact of the Osijek multidisciplinary biopsychosocial program for CLBP compared with standard multimodal care with respect to pain intensity, health-related quality of life, depression, anxiety, and sleep quality using standardized self-assessment questionnaires and a smartwatch. 

## 2. Materials and Methods

### 2.1. Study Design

The study was designed as a prospective, two-arm parallel-group randomized controlled superiority trial with a 1:1 allocation ratio and was conducted from 15 November 2021 to 29 May 2023 at the Department of Anaesthesiology, Resuscitation and Intensive Care, Department of Pain Management of the University Hospital Osijek, following approval from the Ethics Committee of the University Hospital Osijek. No additional long-term follow-up was performed. The trial was retrospectively registered at ClinicalTrials.gov (Identifier: NCT07406711). Patient enrollment began prior to trial registration. The study protocol, prespecified outcomes, and statistical analysis plan remained unchanged after trial commencement. Patients and the public were not involved in the design, conduct, reporting, or dissemination of this study. Respecting the inclusion and exclusion criteria, the study was carried out on 128 participants. To detect a medium effect size in the difference in numerical variables between two groups, with a significance level of 0.05 and power of 0.80, the minimum required sample size is 128 participants (64 per group). To detect a medium effect size in the difference in numerical variables between two repeated measurements, with a significance level of 0.05 and power of 0.90, the minimum required sample size is 108 participants. For this study, the minimum total required sample size is therefore 128 participants (64 per group) (G*Power, ver. 3.1.2). No interim analyses or stopping guidelines were planned or applied. In both participant groups, the first measurement was conducted prior to the four-week period of active treatment, and the second measurement was performed on day 28 of the four-week active treatment period. The standardized self-report instruments used included the Short Form Health Survey (SF-36), the Oswestry Disability Index (ODI 2.1a), the Depression Anxiety Stress Scale (DASS-21), the Numeric Rating Scale (NRS), and the Pittsburgh Sleep Quality Index (PSQI). Basic demographic and social data were also collected. Throughout the four weeks of active treatment, both groups wore a Fitbit Charge 3 (Fitbit, Inc., San Francisco, CA, USA) smartwatch to monitor sleep quality and physical activity (step count). The smartwatch was provided on the first day of study enrollment and worn continuously throughout the four-week treatment period. The devices were registered using an email address, linked to the Fitbit online application, and individually coded for each participant. Smartwatches were connected to the researcher’s mobile phone to enable synchronization, monitoring, and feedback evaluation. The collected data were transcribed into an Excel spreadsheet to enable systematic and detailed analytical processing. Synchronization was required immediately after device setup and twice weekly to ensure proper data upload and transfer to the online application. The smartwatches were charged at the Department for Pain Management. A total of 15 devices were used; after each participant had completed the program, the devices were mechanically cleaned and disinfected, and in cases of wristband damage, the band was replaced with a new one.

### 2.2. Participants

All participants were recruited at the Department for Pain Management of the University Hospital Osijek. Each participant received written information about the study, and all the treatment procedures were additionally explained verbally prior to enrollment. After providing written informed consent, participants were randomly assigned to one of two groups using a random number generator. Participants were randomly assigned in a 1:1 ratio to either the multidisciplinary biopsychosocial program or the multimodal treatment group using a computer-generated random number sequence. Allocation was performed after enrollment. One group was allocated to the multidisciplinary biopsychosocial program for chronic pain management, while the other group received multimodal treatment. Owing to the nature of the multidisciplinary intervention, the blinding of participants and care providers was not feasible. Outcomes were assessed using standardized self-report questionnaires and objective smartwatch monitoring.

The inclusion criteria were as follows: participants with CLBP that significantly affects the degree of disability and/or emotional distress; participants aged 18 to 65 years who agree to participate in the study, confirmed by signing the informed consent; and participants who actively use the Croatian language in written and spoken form. The exclusion criteria were as follows: participants younger than 18 or older than 65 years; participants with acute pain; individuals who had previously completed a multidisciplinary program; refusal to participate; pregnancy; participants with cancer-related pain; presence of a psychotic disorder, uncontrolled psychosis, or moderate to severe cognitive impairment; psychological or psychiatric conditions requiring urgent intervention or preventing the use of cognitive and behavioral teaching methods; participants with symptoms of post-traumatic stress disorder; a high degree of disability that currently exceeds the participant’s functional capacity; presence of extreme fatigue; impaired concentration; low literacy levels that prevent completion of questionnaires and participation in educational components; observed low motivation for active participation; and allergic reactions to metal or silicone. 

### 2.3. Outcome Measurement

The primary outcome was pain intensity measured using the Numeric Rating Scale (NRS). Secondary outcomes included health-related quality of life (SF-36), disability (ODI), psychological distress (DASS-21), subjective sleep quality (PSQI), and objective sleep and physical activity parameters derived from the Fitbit Charge 3.

#### 2.3.1. Numeric Rating Scale (NRS) 

The Numeric Rating Scale (NRS) for pain intensity is a self-report tool consisting of a numbered line ranging from 0 to 10. The left end of the scale (0) indicates no pain, while the right end (10) represents unbearable pain. The numbers between 0 and 10 reflect increasing pain intensity: values from 1 to 3 indicate mild pain, 4 to 6 moderate pain, and 7 to 10 severe pain [34].

#### 2.3.2. Short Form Health Survey (SF-36)

The Short Form Health Survey (SF-36) is one of the most frequently used instruments for evaluating health-related quality of life [35,36]. The Croatian version of the SF-36 was licensed in 1992 at the Andrija Štampar School of Public Health as part of the project Tipping the Balance Towards Primary Healthcare Network [37]. The questionnaire consists of 36 items and eight dimensions. The results are expressed as standardized scores ranging from 0 to 100 for each health dimension, with higher scores indicating better health status. The internal consistency of individual subscales is approximately 0.8 or higher, although occasionally lower for the social functioning scale. Test–retest reliability is somewhat lower on average, ranging from 0.71 to 0.89 [38]. The internal consistency of the validated SF-36 in Croatia ranged from 0.78 to 0.94. Higher scores on individual scales reflect better perceived health in that domain [39]. In the present study, the Cronbach’s alpha coefficient was 0.80, indicating the high reliability of the instrument.

#### 2.3.3. Pittsburgh Sleep Quality Index (PSQI)

The Pittsburgh Sleep Quality Index (PSQI) is a structured self-report instrument used to assess sleep quality and is freely available for educational and non-commercial research purposes. The questionnaire consists of 24 items and includes seven components. Patients assess their sleep quality over the past month. The sum of the seven component scores yields a global score ranging from 0 to 21, with higher scores indicating poorer subjective sleep quality. A PSQI total score greater than 5 is classified as poor sleep quality [40]. The PSQI has been previously validated among chronic pain populations and demonstrates good reliability (Cronbach’s alpha = 0.7) [41]. In this study, the Cronbach’s alpha coefficient was 0.80, indicating high reliability of the instrument.

#### 2.3.4. Oswestry Disability Index (ODI 2.1a)

The Oswestry Disability Index (ODI 2.1a) is a standardized self-report questionnaire designed to assess the level of disability in performing daily activities due to lumbar spine pain [42]. A validated Croatian version of the questionnaire was used [43]. Studies by various authors have shown acceptable internal consistency, with Cronbach’s alpha ranging from 0.71 to 0.90 [42,44,45,46]. Test–retest reliability is high, with intraclass correlation coefficient (ICC) values ranging from 0.83 to 0.99, depending on the time interval between measurements [42,44,45,46,47]. The validated Croatian version demonstrated satisfactory internal consistency (Cronbach’s alpha = 0.84) and test–retest reliability (ICC = 0.94). In this study, the Cronbach’s alpha coefficient was 0.80.

#### 2.3.5. Depression Anxiety Stress Scale (DASS-21)

The Depression Anxiety Stress Scale (DASS-21) is a self-report measure assessing the frequency and severity of negative emotional states—depression, anxiety, and stress—during the preceding week, in both psychiatric and non-clinical populations [48]. A validated Croatian version was used [49]. Each subscale score is obtained by summing the seven item responses, yielding a range from 0 to 21. Higher scores reflect more severe symptoms of depression, anxiety, or stress. Subscale scores are interpreted according to established severity cut-offs, categorized into five levels: normal, mild, moderate, severe, and extremely severe. Cronbach’s alpha values for each subscale are as follows: depression 0.91, anxiety 0.84, and stress 0.90 [48]. The DASS-21 has consistently demonstrated robust psychometric properties across diverse population groups (Cronbach’s alpha > 0.8) [50,51,52]. For the validated Croatian version, Cronbach’s alpha ranges from 0.88 to 0.91 across all three subscales [53]. In this study, Cronbach’s alpha values ranged from 0.88 to 0.94 for all subscales.

#### 2.3.6. Fitbit Charge 3 Smartwatch

Contemporary digitalization has increasingly influenced healthcare delivery [54]. Beyond traditional telemedicine, advances in digital technologies have contributed to the expansion of e-health, enabling access to medical information, self-monitoring, and remote monitoring through connected devices and mobile applications [55]. The growing adoption of e-health is partially driven by the widespread availability of consumer devices used in health-related contexts. In healthcare management, physical activity monitoring may serve both as a self-monitoring tool for patients and as a source of externally collected data for healthcare professionals. Within the framework of shared decision-making, activity monitoring has become increasingly common among individuals with chronic conditions, reflecting a more active patient role in disease management [56]. In addition, data derived from activity-tracking devices may support clinical assessment and contribute to physician–patient communication [54]. Over the past decade, consumer activity trackers including Fitbit devices and smartphone-based accelerometers have gained widespread use [57]. Fitbit is among the most commonly used brands, with 37 million active users in 2022, compared to 9.5 million in 2015 [58]. In the present study, physical activity and sleep-related parameters were monitored using the Fitbit Charge 3 smartwatch. This device allows for continuous monitoring of selected physiological and activity-related parameters, including heart rate, step count, distance, active minutes, energy expenditure, exercise sessions, and sleep characteristics. When synchronized with the accompanying application, data can be exported in PDF or Excel formats for further analysis. Sleep outcomes generated by the device include daily and weekly sleep scores based on aggregated measures of sleep duration and sleep quality. Sleep duration reflects total sleep time and periods of wakefulness, whereas sleep quality is derived from time spent in deep and REM sleep stages. Additional, recovery-related metrics incorporate resting heart rate during sleep and indices of restfulness. The maximum score is 100 points. Most individuals score between 72 and 83. Sleep quality is categorized as excellent (90–100), good (80–89), fair (60–79), or poor (<60) [59]. Physical activity and related parameters were monitored using the Fitbit Charge 3 wearable device. Data obtained from the device were reviewed for completeness and plausibility prior to analysis. Missing values and recordings considered inconsistent or physiologically implausible were excluded according to predefined criteria, and only valid data were included in the final analyses. Non-wear periods and incomplete recordings were identified during data screening and excluded from the analysis to minimize the influence of measurement artifacts.

In addition to the above outcome measures, sick leave was recorded as the number of workdays missed due to chronic low back pain.

### 2.4. Conservative Treatment Methods

#### 2.4.1. Multidisciplinary Biopsychosocial Program for Chronic Pain Management at the Department of Pain Management, University Hospital Osijek

The multidisciplinary program for chronic pain management at the Department for Pain Management of the University Hospital Osijek was implemented on 3 March 2014, modeled after the program of Guy’s & St Thomas’ Hospital in London. Patients were verbally informed about the program during their medical consultation and received detailed written information in a printed brochure. Each patient was given an appointment date, and waiting times generally did not exceed one month. All treatment costs were fully covered by the patient’s basic health insurance. The multidisciplinary biopsychosocial program for chronic pain management is contracted with the Croatian Health Insurance Fund and delivered through the Day Hospital system. One of the main distinctions of this approach compared with the conventional healthcare system is the availability of multiple interconnected professional disciplines in a single setting, with the patient positioned at the center as an equal and active member of the team. The Pain Management Program (PMP) at the Department for Pain Management follows global trends in the treatment of chronic pain among patients for whom previous treatment modalities (pharmacotherapy, physical therapy, minimally invasive and invasive procedures) have proven insufficient. This treatment model was structured as a combination of group and individual therapy involving a multidisciplinary team. The program was based on a multidisciplinary biopsychosocial approach to the management of persistent chronic pain, aiming to educate and engage patients as equal partners in pain management, alongside multimodal treatment methods including pharmacotherapy, exercise, physical therapy, and acupuncture, with individualized work by each team member. The program consisted of the following core components: (1) group-based and individual patient education sessions, (2) group and individual physical therapy (exercise), (3) individualized pharmacological management, (4) individual psychological support, (5) acupuncture and physical therapy modalities, and (6) occupational and nutritional counseling. The multidisciplinary program was delivered across a four-week intervention period, every working day from 8:00 to 14:30, for a group of 7–12 participants throughout the entire year. The daily schedule is structured such that the morning period from 08:00 to 11:30 is allocated to educational sessions and group physical therapy (exercise), followed by a break from 11:30 to 12:00, while the afternoon period from 12:00 to 14:30 is reserved for individual sessions, acupuncture, and physical therapy. The chronic pain management team at the Department for Pain Management, University Hospital Centre Osijek, includes a physician (anesthesiologist–algologist), physiatrist, psychiatrist, psychologist, nutritionist, physiotherapist, occupational therapist, and nurse.

#### 2.4.2. Multimodal Methods in Chronic Pain Management

Transcutaneous electrical nerve stimulation (TENS) was administered using single-channel electrodes on a BTL Smart 4000 device (BTL Industries Inc., Zagreb, Croatia). TENS frequency ranged from 75 to 125 Hz, with pulse widths of 90–100 µs, applied for 10 minutes once daily. Self-adhesive electrodes were placed over the paravertebral musculature in the L2–L5 dermatomes corresponding to the most painful area. Patients were positioned prone or seated. Therapeutic ultrasound was delivered with a BTL Smart 4000 device at a frequency of 1 MHz and an intensity of 0.5–1 W/cm^2^ for three minutes once daily. The ultrasound was applied continuously using conductive gel over the paravertebral musculature in the L2–L5 dermatomes in order to achieve a thermal effect. Patients were positioned prone or seated. Magneto therapy was applied using a BTL 4000 device equipped with electromagnetic therapy components and flat electrodes. A pulsed magnetic field up to 100 Hz, with an intensity of 31 mT/10, was administered for 10 minutes once daily; as this application mode is commonly used in chronic musculoskeletal pain due to its favorable safety profile and suitability for repeated daily treatment. Patients lay in the supine position, with a 60 × 25 cm flat electrode placed under the lumbar spine. For acupuncture therapy, a quiet and relaxing environment with soft background music was provided, ensuring patient privacy. Patients lay in the prone position during the 20 min treatment. Single-use, sterile stainless-steel needles were applied; needles were available in two lengths and diameters (0.20 × 15 mm and 0.30 × 30 mm) with silicone-coated handles.

### 2.5. Statistical Methods

All collected categorical data are presented as absolute and relative frequencies, while numerical data are presented exclusively as medians and interquartile ranges, and additionally with overall ranges when necessary, as their distributions did not follow a normal Gaussian distribution. The Shapiro–Wilk test was used to assess normality. Standard statistical methods were employed for data analysis. Categorical variables within and between groups were compared using the chi-square test. Differences between two independent groups of numerical variables were assessed using the nonparametric Mann–Whitney U test, while differences between repeated measurements were evaluated using the nonparametric Wilcoxon test. Associations between pairs of numerical variables were assessed using Spearman’s nonparametric rank correlation test (rho). Collected data were first entered into a spreadsheet in MS Office Excel (version 2016, Microsoft Corp., Redmond, WA, USA). Statistical analyses were performed using MedCalc (version 22.009, MedCalc Software Ltd., Ostend, Belgium) and IBM SPSS Statistics (version 24.0.0.0, IBM Corp., Armonk, NY, USA), with a predefined significance level of α = 0.05. All P-values were two-tailed. All randomized participants were included in the final analysis according to the intention-to-treat principle. No missing outcome data were observed; therefore, no imputation methods were required. No prespecified subgroup or sensitivity analyses were conducted.

### 2.6. Ethical Principles

Approval for the study was obtained from the Committee for Ethical and Professional Issues of the University Hospital. Before participation, all participants were informed about the aim and specific characteristics of the study and received a written Participant Information Sheet as well as an Informed Consent Form, which was additionally explained verbally. By signing the consent form, participants voluntarily agreed to take part in the study. Participants were ensured anonymity during and after the study; therefore, the data obtained from questionnaires and the smartwatch cannot be linked to individual participant identifiers.

## 3. Results

### Participant Flow

**Figure 1:**
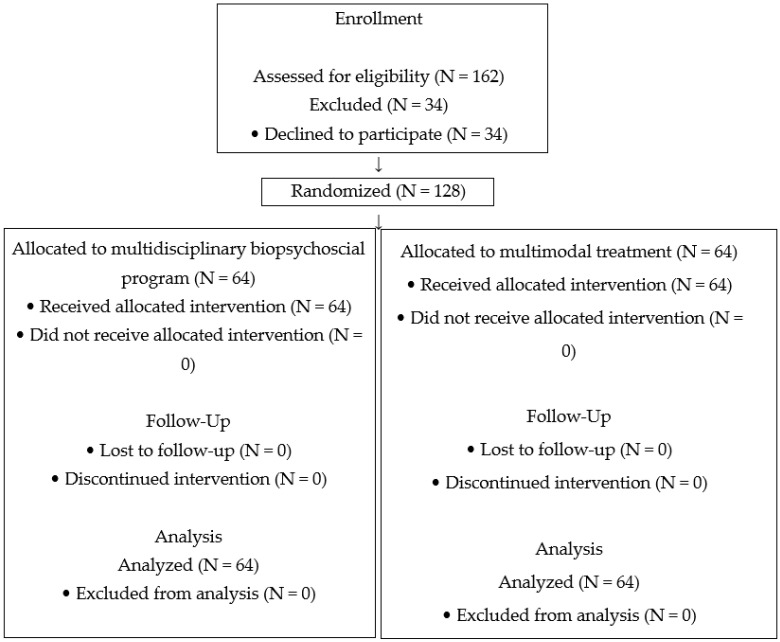
CONSORT flow diagram of participants.

A total of 162 patients were assessed for eligibility, of whom 34 declined participation due to perceived additional burden. Consequently, 128 participants were enrolled and randomly assigned in a 1:1 ratio to the multidisciplinary biopsychosocial group (n = 64) or the multimodal treatment group (n = 64). No losses or exclusions occurred after randomization. All participants received the allocated intervention as planned, with no protocol deviations, and were included in the final analysis. Participants continued their usual prescribed pharmacological therapy throughout the study, as determined by their treating physicians. A participant flowchart of the study is presented in Figure 1 (for additional details, see the Supplementary Materials, Table S1) [60]. 

The median age of all participants was 52 years, with an interquartile range of 45 to 58.5 years and a total range of 29 to 65 years. A total of 128 participants took part in the study, with a significantly higher proportion of women (Chi-square test, *p* < 0.001). Participants were significantly more likely to be married and living with a partner (Chi-square test, *p* < 0.001), as well as living with a partner and children (Chi-square test, *p* < 0.001). They more frequently resided in urban areas (60.2%) and were significantly more likely to have a secondary level of education (Chi-square test, *p* < 0.001). However, fewer than half of the participants were employed, and more than one quarter were on medical leave (Chi-square test, *p* < 0.001) (Table 1).

Half of the participants reported taking medication regularly, while slightly fewer than one third used medication as needed (Chi-square test, *p* = 0.02). A significant majority (Chi-square test, *p* = 0.02), amounting to two thirds of respondents, had taken medical leave due to low back pain within the past five years. Most participants were non-smokers (Chi-square test, *p* = 0.001); however, a large majority either did not exercise or exercised only once per week (Chi-square test, *p* < 0.001). Half of the participants reported having other chronic conditions and one or more comorbidities (Table 2).

The body mass index (BMI) of participants was calculated, yielding a median value of 27.0 (interquartile range 24.2 to 30.4). The average duration of pain prior to initiation of either treatment program was seven years (median; interquartile range 3.5 to 12 years). Among the 65.6% of participants who had taken sick leave in the past five years, the median number of sick leave was episodes three (interquartile range 2 to 5). At baseline, participants were divided into two groups according to the type of rehabilitation program: a multidisciplinary program or a multimodal treatment program. Accordingly, demographic characteristics and baseline health status between the two study groups. No statistically significant differences were observed in mean age, pain duration, BMI, or frequency of sick leave episodes within the past five years. However, a tendency toward baseline differences was observed, as participants treated with the multimodal approach had a slightly shorter pain duration (borderline statistical significance) and reported half as much sick leave within the past five years compared with those treated using the multidisciplinary approach (Table 3).

There were no significant differences between the two groups with respect to other categorical demographic characteristics. The proportions of men and women were similar across both groups, as were the proportions of participants living with a partner versus living alone. Likewise, the distribution of participants residing in urban versus rural areas was comparable, and the groups did not differ in terms of educational attainment or employment status (Table 4).

The two participant groups did not differ significantly with respect to sick leave. However, baseline differences between groups were observed in several other baseline health characteristics. Participants who were later treated with the multidisciplinary approach—that is, those assigned to the first group—were significantly more likely to take medication regularly (Chi-square test, *p* = 0.002), to engage in exercise (Chi-square test, *p* = 0.04), and to have other chronic diseases (Chi-square test, *p* = 0.003), as well as one or more comorbidities (Chi-square test, *p* = 0.01) (Table 5).

The following section presents the results of the analysis of potential differences in baseline values of all measured variables, including pain, disability, health-related quality of life, anxiety, depression, stress, and sleep quality.

Baseline differences between groups were observed, but no significant differences were found in baseline pain intensity (measured using the standard NRS), except for the pain subscale of the SF-36 questionnaire. Participants who were later treated using the multidisciplinary approach reported significantly higher pain levels on this subscale (Mann–Whitney U test, *p* = 0.02). Overall, participants in this first group demonstrated significantly poorer outcomes (Mann–Whitney U test, *p* < 0.04) across most domains of the SF-36 health-related quality-of-life questionnaire. The two participant groups differed significantly in baseline disability levels (ODI) (Mann–Whitney U test, *p* = 0.004), with those later treated using the multidisciplinary approach (the first group) exhibiting significantly worse scores. This group also showed a significantly higher level of anxiety as measured by the DASS-21 questionnaire (Mann–Whitney U test, *p* = 0.04). The groups did not differ significantly across most individual parameters of the PSQI sleep quality questionnaire, except for sleep duration, where the first group reported significantly shorter sleep on average (Mann–Whitney U test, *p* = 0.01). Additionally, the overall PSQI sleep quality score was significantly higher (Mann–Whitney U test, *p* = 0.02) in the first group, indicating poorer sleep quality among participants who were later treated with the multidisciplinary approach (Table 6).

All variables assessing pain, disability, health-related quality of life, anxiety, depression, stress, and sleep quality were measured at baseline and again upon completion of the multidisciplinary or multimodal chronic pain treatment program. The following section presents the results of comparing baseline and post-treatment values for participants treated with the multidisciplinary biopsychosocial approach. Significant within-group improvements were observed (Wilcoxon test, *p* < 0.01) in pain intensity measured by the standard NRS, as well as in all scales and subscales of the administered questionnaires, with the exception of the PSQI subscale assessing difficulties performing daily tasks due to insufficient sleep. For this subscale, the results indicated partial improvement, although the change reached only borderline statistical significance (Wilcoxon test, *p* = 0.06) (Table 7). Overall, the multidisciplinary biopsychosocial group showed consistent within-group improvement across the majority of evaluated outcomes following completion of the program.

For the second group of participants—that is, patients treated with the multimodal approach—all baseline and post-treatment values were likewise compared. The comparison of baseline and post-treatment values derived from the questionnaires assessing pain, disability, health-related quality of life, depression, anxiety, and stress, and sleep quality indicated significant within-group improvement (Wilcoxon test, *p* < 0.03) among participants treated with the multimodal approach (Table 8). However, no significant improvement was observed in depression or anxiety as measured by the DASS-21 questionnaire, nor in difficulties performing daily activities due to insufficient sleep or in the use of pharmacological agents, as measured by the PSQI sleep quality questionnaire. Overall, participants treated with the multimodal approach demonstrated measurable within-group improvements in several evaluated parameters, although improvements were not observed consistently across all outcome domains (Table 7 and Table 8). Participants in the second group demonstrated significant improvement in most evaluated parameters following multimodal treatment. However, their improvements did not extend across all domains to the same extent as in the first group; i.e., those treated with the multidisciplinary biopsychosocial approach.

The following section presents an exploratory comparison of unadjusted change scores observed at the end of each treatment type. The analysis was based on absolute differences between post-treatment and baseline values for all examined variables. The results show that changes in several outcome measures tended to be larger among participants treated with the multidisciplinary biopsychosocial approach. Improvements in physical functioning were larger and reached borderline statistical significance (Mann–Whitney U test, *p* = 0.05), while the improvement in energy levels was significantly greater (Mann–Whitney U test, *p* = 0.04), both assessed using the SF-36 health-related quality-of-life questionnaire (Table 9). Consistent with the within-group findings reported above (Table 7 and Table 8), between-group comparisons of change scores indicated larger reductions in depression and anxiety levels (Mann–Whitney U test, *p* < 0.001), as well as a greater reduction in the reported use of pharmacological sleep aids (Mann–Whitney U test, *p* = 0.04) among participants treated with the multidisciplinary approach (Table 9). These findings should be interpreted cautiously, in light of the baseline differences between groups and the unadjusted nature of the analyses (Table 9).

No significant between-group differences were observed in objectives, e.g., sleep quality scores measured by the Fitbit smartwatch, or in physical activity levels assessed by daily step count (Table 10).

**Figure 2:**
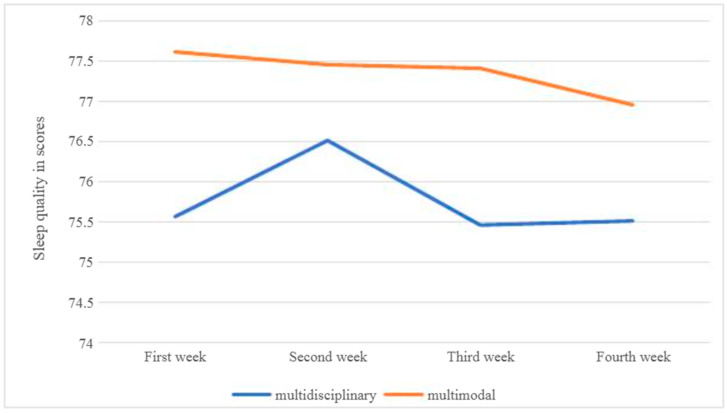
Weekly distribution of sleep quality scores for each participant group.

Similarly, differences in the weekly distribution of sleep quality scores within each participant group were minimal (Figure 2).

**Figure 3:**
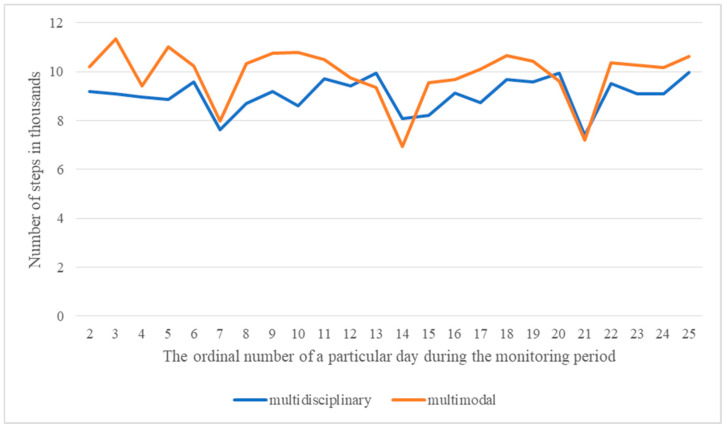
Daily distribution of step counts during the observation period for each participant group.

The distribution of daily step counts recorded by the Fitbit smartwatch was also comparable between the two groups, with a consistent decrease observed on Sundays in both groups (Figure 3).

Within each participant groups, weak negative associations was identified between sleep quality scores and daily steps counts. These associations reached statistical significance in one group (Spearman correlation, rho = –0.234, *p* = 0.04) and borderline significance in the other (Spearman correlation, rho = –0.199, *p* = 0.06). Given the small effect sizes and the observational nature of these analyses, these findings should be interpreted cautiously, as they may reflect the influence of unmeasured confounding factors rather than a direct relationship between physical activity and sleep quality (Table 11).

No adverse events or intervention-related harms were reported in either group during the four-week intervention period. No prespecified subgroup or sensitivity analyses were conducted. Correlation analyses were exploratory in nature.

## 4. Discussion

This randomized controlled trial demonstrated that participants with chronic low back pain who underwent the Osijek multidisciplinary biopsychosocial program experienced greater improvements in quality of life compared with those receiving standard multimodal treatment. Given the differences in the structure and implementation of multidisciplinary programs across clinical settings, the aim of this study was to assess the relative impact of the Osijek multidisciplinary program for chronic low back pain (CLBP) compared with standard multimodal care with respect to pain intensity, disability, health-related quality of life, depression, anxiety, stress, and sleep quality using standardized self-assessment questionnaires and a smartwatch. At baseline, a total of 128 participants were included in the study and randomly allocated to a multidisciplinary biopsychosocial program or a multimodal treatment program. Baseline demographic characteristics and health status were compared between the two groups. No statistically significant differences were observed in mean age, body mass index, or pain duration prior to therapy initiation. Although the difference did not reach statistical significance, participants treated with the multimodal approach tended to report a shorter pain duration and approximately half as many sick leave episodes within the past five years compared with those treated using the multidisciplinary approach. In both groups, there was a significantly higher proportion of women. The median age of all participants was 52 years, with an interquartile range of 45 to 58.5 and an overall range of 29 to 65 years. Similar participant characteristics have been reported in previous studies [61,62], and the average age of the sample is similar to that of the general adult population in the Republic of Croatia [63], suggesting that the study population is broadly representative in this respect. Regarding pharmacotherapy, half of the participants reported regular medication use, slightly less than one-third reported taking medication as needed, and slightly less than one-quarter reported not taking medication at all. Given that recommended pharmacotherapy in chronic pain generally implies continuous medication intake, taking analgesics only as needed may indicate suboptimal adherence, which places this group closer to participants who do not take medication regularly. This observation is consistent with previous findings showing that, although pharmacotherapy represents a cornerstone of chronic pain management [64], a substantial proportion of prescribed medications—approximately 40%—are not taken as prescribed [65]. However, the present study did not directly assess reasons for non-adherence, and alternative explanations, such as individual pain fluctuations or concerns about medication use, cannot be excluded. Nevertheless, such a high rate of inadequate pharmacotherapy adherence may be partly explained by the complexity of chronic pain and its impact on the whole person, considering the results of this study that indicate prolonged pain and high pain intensity. In this study, in both participant groups, the majority of participants did not exercise or exercised only once per week. The previous literature indicates that regular exercise represents a key component of effective CLBP management [66,67]. Current guidelines for CLBP treatment recommend an active lifestyle, patient education, and exercise therapy [67], emphasizing the importance of adapting exercise to individual patients through a multimodal approach [68] while also considering potential fear of pain and movement [69]. In the present study, limited engagement in regular exercise may be related to the generally high average pain intensity, which may act as a barrier to physical activity due to fear of increased pain clinical deterioration, or potential injury. Additionally, half of the participants reported other chronic diseases and one or more comorbidities. In their study, Moses-Hampton et al. found that patients with CLBP have a higher number of comorbidities compared to the control group [70], which may further limit participation in regular exercise. Participants in this study had a median Body Mass Index (BMI = kg/m^2^) of 27.0 (interquartile range 24.2–30.4), and were classified as overweight according to World Health Organization (WHO) [71], a factor that may also influence physical activity levels in this population. Due to inconsistent findings regarding the causal relationship between obesity and poor sleep [72,73], it may be important to consider this potential association between overweight, sleep quality, pain intensity, and HRQOL in patients with CLBP. The average pain duration among participants was seven years prior to enrollment in either the multidisciplinary program or multimodal treatment. However, patients treated with the multimodal approach had a slightly shorter pain duration. Considering the definition of chronic pain, it is necessary to underscore the presence of long-lasting CLBP in both participant groups. Pain duration has been identified as an important predictor of treatment efficacy and has been shown to negatively influence treatment outcomes even within a multidisciplinary approach, with greater improvements generally observed in patients with shorter pain duration [16]. In the context of the present study, the prolonged pain duration observed among participants highlights the complexity and challenges associated with the management of chronic pain, particularly CLBP. Despite ongoing research and advances in treatment techniques [74], many chronic pain conditions remain insufficiently understood and managed, which may contribute to persistent pain and its negative impact across multiple domains of patients’ lives [75,76]. 

Participants treated with the multidisciplinary biopsychosocial program showed significant improvements in all observed parameters upon the completion of the program for the treatment of CLBP. These results are consistent with the existing literature, which widely recognizes this approach as the gold standard for chronic pain management [14,28,77]. 

Before the intervention, participants were classified as being in the severe pain category. After the intervention, pain intensity was reduced, with participants reaching the moderate pain category [34]. It should be emphasized that the goal of the program is not necessarily focused solely on pain intensity reduction but, rather, on restoring the patient to a more functional level [32]. Furthermore, the results demonstrated significant improvement across all subscales of the questionnaire assessing HRQoL, supporting the positive impact of the treatment approach and aligning with the literature indicating that multidisciplinary pain treatment improves HRQoL life in most patients [78,79]. The findings of this study also suggest that participants reported lower self-perceived physical health compared with mental health, as assessed by the SF-36 questionnaire, a pattern consistent with trends observed in comparable research [80,81]. Specifically, the results suggest that participants rated the domains of role limitations due to physical problems, bodily pain, physical functioning, and general health perception as the lowest. Limitations in physical functioning may contribute to reduced time spent at work or difficulties in performing planned activities. In a study conducted by Martinec et al., patients with rheumatoid arthritis reported the poorest outcomes in the domains of physical limitations and functioning, while the most favorable outcomes were observed in mental health [82], which partially aligns with the findings of the present study. Studies conducted in other European countries have reported similar findings indicating poorer self-assessment of both physical and mental health among patients with LBP [83,84]. Furthermore, in addition to poorer ratings of physical functioning compared with mental health, bodily pain was identified as the lowest-rated subscale. Considering high pain intensity, limitations due to physical health, bodily pain, and impaired physical functioning, a high degree of disability would be anticipated, placing participants in the severe disability category according to the Oswestry Disability Index (ODI) questionnaire. This ODI category indicates that pain represents a primary problem for participants in the present study. Previous research has also demonstrated the negative impact of pain intensity on HRQoL [20,85], as well as the influence of disability on quality of life [20]. After the intervention, significant improvements were observed across all these subscales, that is, in the overall dimension of the questionnaire related to physical health.

In this study, participants also reported impaired overall mental health. These findings are consistent with the literature, indicating that chronic pain can affect all aspects of a patient’s life, including physical and emotional health. In this context, a multidisciplinary biopsychosocial approach appears appropriate, as it addresses both physical and psychological dimensions of chronic pain. Following participation in the multidisciplinary biopsychosocial program, significant improvements were observed across all mental health subscales. These findings are consistent with previous research reporting beneficial effects of multidisciplinary approaches on reducing disability [78]. In this study, results obtained using the DASS-21 questionnaire indicated the presence of mild depression and anxiety, while stress levels were within the normal range. These findings are consistent with the literature, which highlights psychological factors as an important domain in CLBP when assessing treatment efficacy [86]. Furthermore, Dimitrijevic and Knez reported a moderate positive correlation between anxiety, depression, and pain intensity in patients with chronic pain, as well as a reduction in anxiety, depression, and pain intensity following participation in a multidisciplinary program chronic pain treatment program [87]. After the intervention, all DASS-21 subscales showed significant improvement. These findings are consistent with the literature, which emphasizes that the effective treatment of CLBP requires a multidisciplinary, holistic approach to the management of psychological distress [88]. Sleep quality was assessed using the PSQI questionnaire, with the results indicating poor sleep quality [40]. These findings are consistent with previous research reporting an association between sleep quality and pain intensity, whereby poor sleep quality is linked to increased pain intensity in patients with CLBP [89]. Furthermore, adults with CLBP who experience sleep disorders have been shown to have a 13–60 % lower likelihood of recovery over a ten-year follow-up period compared to those without sleep disorders [90]. Following the intervention, improvement was observed across all PSQI subscales, except for the subscale assessing difficulties in performing daily activities due to poor sleep. Further analysis indicated partial improvement in this domain, reaching borderline statistical significance. Although significant improvements were observed after the intervention, overall PSQI scores continued to classify sleep quality as poor. Nevertheless, these findings are consistent with the literature, suggesting that multidisciplinary pain management programs have the potential to improve sleep quality [91,92].

In participants included in multimodal treatment, pain intensity, assessed using the NRS, was classified as severe. In addition, baseline results indicate poorer outcomes in the domain of physical health compared with mental health, which is consistent with previous findings [80,81,82]. The degree of disability assessed using the ODI questionnaire placed participants in the category of moderate disability, which is in line with previous research, identifying age, pain intensity, and depression as important factors associated with disability in patients with CLBP [93]. In the present study, pain intensity may represent a relevant factor associated with disability, given that the other examined factors did not show clinical significance. Furthermore, impaired physical health as assessed by the SF-36 questionnaire, with role limitations due to physical health being the lowest-rated dimension, is consistent with the presence of functional limitations in this population. The DASS-21 indicates normal levels of depression, anxiety, and stress among participants. The finding is not fully consistent with the previous literature and may be interpreted in the context of long-term adaptation to chronic pain, whereby individuals develop coping or defense mechanisms that allow more effective functioning despite prolonged CLBP. Such adaptive mental mechanisms are described as automatic processes that help regulate emotional responses and mitigate the harmful effects of pain as described by Vaillant [94]. Participants subjectively assessed their sleep quality as impaired, which is consistent with the literature, which shows an association between sleep quality and pain intensity, with poor sleep quality being linked to increased pain intensity in patients with CLBP [89]. Following multimodal treatment, significant improvements were observed across most assessed parameters; however, no significant changes were detected in depression and anxiety levels as measured by the DASS-21 questionnaire. Furthermore, no significant improvement was observed in difficulties performing daily activities due to poor sleep, nor was there a significant reduction in the use of sleep medication as measured by the PSQI questionnaire. In the context of the present sample, these findings suggest that, despite overall improvement, it is difficult to disentangle the specific contribution of pharmacotherapy within the multimodal treatment approach, given that most participants at baseline were either not taking the prescribed medication or were taking it on an as-needed basis. The lack of justification for this assumption may be related to the fact that during and after treatment, it remained unclear whether patients began taking their prescribed therapy regularly. Despite high pain intensity, the substantial limitations due to physical health, and moderate disability prior to treatment, it cannot be determined whether participants consistently adhered to analgesic therapy recommendations. A self-initiated change in adherence behavior may be limited, particularly in the absence of additional education during multimodal treatment. In addition, participants were included in acupuncture as part of chronic pain management, an approach that has been suggested in the literature as being particularly suitable for patients who prefer to avoid analgesic use [95]. Although this assumption is only indirectly supported by the available data, it cannot be entirely dismissed. Moreover, the literature indicates that even patients who do not consistently follow physicians’ instructions may experience reductions in pain intensity [96]. Accordingly, it is possible that factors not directly assessed in the present study contributed to pain reduction, and the relatively small sample size should be considered when interpreting these findings. With regard to physical therapy modalities, proposed approaches for CLBP include certain electrotherapy interventions (e.g., laser therapy, interferential therapy, ultrasound) and transcutaneous electrical nerve stimulation (TENS). However, the literature data indicate that these are not recommended in National Institute for Health and Care Excellence (NICE) or European guidelines for CLBP due to a lack of supporting evidence and insufficient data on cost-effectiveness. According to NICE guidelines, the only procedure recommended is acupuncture, whereas European guidelines also allow percutaneous electrical nerve stimulation (PENS) and neuro-reflex therapy [97]. Liu et al. reported that acupuncture was clinically more effective than no treatment in terms of pain relief and functional improvement in the short term, and that acupuncture as an adjunct to conventional therapy provided a clinically meaningful short-term reduction in pain and improvement in functional limitations in patients with CLBP [98]. In the present study, differences in most examined variables were numerically greater among patients treated with the multidisciplinary biopsychosocial approach. In particular, patients treated with this approach demonstrated greater improvements in depression and anxiety compared with those receiving the multimodal treatment, which should be interpreted in the context of higher baseline anxiety levels in this group. More pronounced improvements in physical functioning and energy/vitality may be partially explained by the use of individualized exercises during the multidisciplinary program. This interpretation should be considered in the context of baseline differences between groups, as participants in the multidisciplinary group were significantly more likely to engage in exercise at baseline. According to data from the meta-analysis by Searle et al., exercise has a small but significant benefit in the treatment of CLBP and is more effective than conservative therapies such as electrotherapy procedures, while strength/resistance training, coordination/stabilization exercises, and combined exercise programs show greater effectiveness than conservative approaches. Furthermore, in the context of significant reductions in anxiety and improvements in sleep quality, more pronounced improvement may be possible, supporting the potential for increased physical activity, as described in the literature [99], which in this context refers to overall daily physical activity rather than structured physical exercise. It is also possible that the significant reduction in the use of sleep medication observed among patients treated with the multidisciplinary program contributed to the overall improvement in the assessed domains and should therefore not be overlooked. However, this interpretation, as well as the clinical significance of the observed improvements, should be approached with caution. Patients treated with the multimodal approach showed normal anxiety; although sleep quality improved in both groups, it remained classified poor before and after treatment. Karaman et al. emphasize that sleep quality and fatigue are frequently comorbid with pain and suggested that multiple mechanisms may underlie the benefits of multidisciplinary treatment approaches [100]. The PSQI questionnaire results suggest that patients included in multimodal treatment were not taking pharmacotherapy to facilitate sleep, despite reporting poor sleep quality. The observed improvement may reflect the development of more effective, non-pharmacological strategies for sleep management within the context of multidisciplinary care, in line with conclusion reported by Karaman et al. Furthermore, patients treated with the multidisciplinary biopsychosocial approach demonstrated greater improvements in depression and anxiety. These findings should be interpreted in the context of baseline differences between groups, but remain noteworthy given the multidimensional impact of CLBP and the rationale supporting a biopsychosocial treatment framework. 

A component of the Osijek multidisciplinary program includes cognitive–behavioral therapy (CBT) and acceptance and commitment therapy (ACT). These methods primarily focus on the acquisition of skills that facilitate more effective coping with chronic pain and aim to improve daily functioning. The literature suggests that various therapeutic techniques used within CBT and ACT may contribute to reductions in anxiety, depression, and stress, thereby improving overall quality of life in patients with CLBP [101]. Although the obtained results indicate that both treatment approaches were associated with significant improvements, participants in the first group demonstrated significant improvements across all observed parameters by the end of the Osijek multidisciplinary biopsychosocial program for CLBP. In both groups of participants, no significant difference were observed in sleep quality, assessed by the Fitbit smartwatch (measured in points), or in the level of physical activity, assessed by the average daily step count and also measured using the Fitbit smartwatch. Differences in the weekly distribution of sleep quality scores within each group were minimal, which is consistent with the subjective assessment obtained using the PSQI questionnaire. Specifically, both groups of participants rated their sleep quality as poor before the intervention and, although statistically significant improvements were observed, overall sleep quality remained classified as poor after the intervention. Furthermore, when comparing sleep quality assessed using the PSQI questionnaire obtained from the smartwatch, both measures pointed in the same direction, indicating poor or low-quality sleep. The persistence of poor sleep quality may be related to the coexistence of excessive body weight, pain intensity, the long-term experience of chronic pain, and impaired mental health observed among participants in this study. 

Quality-of-Life Technologies (QoLT) provide real-time feedback through smart devices that individuals can use in their daily lives to assess and potentially improve health and well-being [102]. Many sleep-tracking devices appear to meet the basic objectives of QoLT by enabling users through mobile applications or wearable technologies. Personal sleep tracking technologies may contribute to the improved quality of life by increasing awareness of sleep behavior and facilitating the exploration of associations with other quality-of-life indicators and potential sleep related problems. However, data collection and interpretation can be challenging, as they often require specific expertise in sleep technology and sleep monitoring [103]. The distribution of daily step count recorded by the Fitbit smartwatch wristband per day largely overlapped between the two participants groups. A decrease in the number of steps was consistently observed on Sundays, which may reflect greater engagement in passive rest on that day. In both groups, a significant but weak and negative correlation was found between sleep quality and the number of steps taken per day. This indicates that participants with poorer sleep quality, as assessed by the Fitbit smartwatch tended to accumulate a higher number of daily steps. One possible explanation for this pattern may relate to nocturnal activity, as some participants reported difficulties remaining in a lying position due to pain, which prompted getting up and walking during the night. In the study by Widmann et al., the authors noted that wearing fitness wristbands can enhance patients’ sense of active involvement in managing chronic pain, while in clinical settings such devices enable objective and continuous monitoring of progress [104]. In the present study, the use of a smartwatch aimed to partially objectify the assessment of physical activity and sleep quality. When interpreting these findings, the subjective nature of standardized questionnaires should be considered. Although their use is well justified, questionnaire-based assessments rely on participants’ self-reports and may therefore be influenced by memory, cognitive processes, socially desirable responding, and other response biases under psychological influence. In addition, potential limitations and subjective bias related to clinical evaluators should also be acknowledged [105]. Likewise, the literature emphasizes that researchers must be aware of limitations associated with the accuracy and applicability of wearable devices and algorithms, as well as issues related to data quality and interpretation [106].

## 5. Limitations

This study has several limitations that should be considered when interpreting the findings. First, the sample was drawn from a single clinical center—the Department of Pain Management at the University Hospital Osijek—which may limit the generalizability of the results to broader or more diverse patient populations. Second, baseline imbalances between the study groups were observed, which may limit the interpretation of differences in outcomes between the multidisciplinary biopsychosocial and multimodal treatment approaches. Although these baseline differences were considered in the discussion, they reduce the ability to attribute observed between-group differences solely to the effects of the interventions. In addition, differences in the intensity and composition of interventions across study arms may have contributed to performance bias and should be considered when interpreting the results. Furthermore, the study relied heavily on self-reported measures, which may be susceptible to response bias, especially for subjective constructs such as pain intensity, stress, anxiety, and sleep quality. In addition, the DASS-21 questionnaire was used to assess the severity of depressive, anxiety, and stress-related symptoms rather than to establish clinical diagnoses, and it does not allow differentiation between symptoms related to chronic low back pain and those that may have pre-existed the condition. The use of the Fitbit Charge 3 smartwatch provided objective data on sleep and physical activity; however, wearable devices have known limitations in accurately capturing sleep architecture and differentiating activity types, which may have influenced the precision of the objective measurements. The relatively short duration of the intervention (four weeks) restricts conclusions about long-term treatment effects. The weak negative correlation identified between sleep quality and step count should also be interpreted cautiously, as the study design does not allow for causal inferences. In addition, step count reflects overall daily physical activity rather than structured physical exercise, and therefore changes in step count should not be interpreted as changes in exercise behavior. Finally, potential confounding factors—such as medication use, comorbidities, or lifestyle variables—were not controlled for comprehensively, which may have impacted the observed outcomes. Taken together, these limitations highlight the need for future large-scale randomized controlled trials with more diverse samples and longer follow-up periods to further validate and extend these findings.

## 6. Implications for Practice

Findings from this prospective study suggest several important implications for clinical practice in the management of chronic low back pain (CLBP). First, the observed improvements in pain intensity, disability, stress, anxiety, sleep quality, and health-related quality indicate the potential value of multidisciplinary biopsychosocial programs within routine care. Clinicians may consider integrating coordinated interventions—such as physical therapy, psychological support, patient education, and structured activity planning—rather than relying exclusively on conventional multimodal treatment approaches. The present findings suggest that short-term, structured biopsychosocial interventions may be associated with clinically meaningful benefits over a four-week period, indicating that such programs could even be feasible in demanding clinical settings. The use of objective monitoring tools, such as wearable devices, may support clinical decision-making by providing additional information on sleep and activity patterns; however, their technological and interpretative limitations should be taken into account. Given the weak negative association observed between sleep quality and physical activity, a holistic perspective may be warranted when addressing patient sleep disturbances in patients with CLBP, as activity levels alone may not fully explain sleep-related difficulties. Accordingly, sleep hygiene education and behavioral strategies may represent useful components of comprehensive management, when tailored to individual patient needs. Finally, the study’s limitations highlight the importance of individualized and flexible care. Factors such as comorbidities, medication use, and lifestyle characteristics should be carefully considered when interpreting outcomes and planning treatment. Until more robust evidence from larger-scale randomized controlled trials becomes available, multidisciplinary biopsychosocial approaches may be incorporated into patient-centered care, with ongoing evaluation and adjustment based on treatment response.

## 7. Conclusions

This study suggests that a multidisciplinary biopsychosocial program may offer greater improvements in health-related quality of life, psychological well-being, and sleep quality in patients with chronic low back pain compared to a multimodal conservative approach. Physiotherapy within the multidisciplinary program emphasized structured exercise and individualized functional interventions, while the multimodal treatment primarily involved pharmacotherapy and conventional physical therapy modalities. Observed increases in daily physical activity were associated with improvements in anxiety and sleep, though causal relationships cannot be inferred. Despite baseline imbalances, this randomized controlled trial supports the integration of a structured multidisciplinary biopsychosocial program into chronic low back pain management within a real-world clinical setting. Further large-scale and multicenter studies with extended follow-up may help to confirm the generalizability and long-term sustainability of these findings.

## Figures and Tables

**Table 1:** Sociodemographic characteristics of the participants.

	Variable	N (%)	*p* *
Gender	Male	32 (25.0)	<0.001
	Female	96 (75.0)	
Marital status	Married	92 (71.9)	<0.001
	Divorced	18 (14.1)	
	Non-marital cohabitation	2 (1.6)	
	Widowed	3 (2.3)	
	Single	13 (10.2)	
Cohabitation	Lives alone	19 (14.8)	<0.001
	With spouse	34 (26.6)	
	With parents	2 (1.6)	
	With children	10 (7.8)	
	With spouse and children	54 (42.2)	
	Other	9 (7.0)	
Place of residence	City	77 (60.2)	0.10
	Village	51 (39.8)	
Education degree	Primary school	13 (10.2)	<0.001
	High school	99 (77.3)	
	Bachelor	9 (7.0)	
	Master	7 (5.5)	
Working status	Employed	54 (42.2)	<0.001
	Unemployed	15 (11.7)	
	Retired	20 (15.6)	
	On medical leave	34 (26.6)	
	Disability pension	5 (3.9)	
In total		128 (100.0)	

* Chi-square test.

**Table 2:** Baseline health status of the participants.

	Variable	N (%)	*p* *
Medication use	Yes	64 (50.0)	0.02
	No	28 (21.9)	
	As needed	36 (28.1)	
On sick leave	Yes	84 (65.6)	0.01
	No	44 (34.4)	
Physical exercise	Does not exercise	53 (41.4)	<0.001
	Exercises once per week	20 (15.6)	
	Exercises twice per week	25 (19.5)	
	Exercises three times per week	10 (7.8)	
	Exercises four or more times per week	6 (4.7)	
	Exercises daily	14 (10.9)	
Other chronic diseases	Yes	65 (50.8)	0.90
	No	63 (49.2)	
Comorbidity	No	62 (48.4)	0.003
	One	46 (35.9)	
	Two and more	20 (15.6)	
In total		128 (100.0)	

* Chi-square test.

**Table 3:** Comparison of the two participant groups according to selected demographic characteristics.

	Median (25–75 %)		*p* *
	Multidisciplinary	Multimodal	
Age	54.0 (46.0–58.3)	50.0 (43.0–58.8)	0.19
Duration of pain in years	9.0 (5.0–14.3)	6.0 (2.0–11.8)	0.054
BMI	27.5 (24.2–31.3)	26.6 (24.4–30.2)	0.51
Number of sick leave in the past five years	4.0 (2.0–8.0)	2.0 (1.0–5.0)	0.07

* Mann–Whitney U test.

**Table 4:** Comparison of additional demographic characteristics between the two participant groups.

	Variable	N (%)		*p* *
		Multidisciplinary	Multimodal	
Gender	Male	18 (28.1)	14 (21.9)	0.42
	Female	46 (71.9)	50 (78.1)	
Marital status	Married, Non-marital cohabitation	47 (73.4)	47 (73.4)	>0.99
	Divorced, Widowed, Single	17 (26.6)	17 (26.6)	
Cohabitation	Lives alone	9 (14.1)	10 (15.6)	0.55
	With spouse	19 (29.7)	15 (23.4)	
	With parents	7 (10.9)	5 (7.8)	
	With children	23 (35.9)	31 (48.4)	
	With spouse and children	6 (9.4)	3 (4.7)	
Place of residence	City	38 (59.4)	39 (60.9)	0.86
	Village	26 (40.6)	25 (39.1)	
Education degree	Primary school	8 (12.5)	5 (7.8)	0.81
	High school	49 (76.6)	50 (78.1)	
	Bachelor	4 (6.3)	5 (7.8)	
	Master	3 (4.7)	4 (6.3)	
Working status	Employed	21 (32.8)	33 (51.6)	0.19
	Unemployed	9 (14.1)	6 (9.4)	
	Retired	15 (23.4)	10 (15.6)	
	On medical leave	19 (29.7)	15 (23.4)	
In total		64 (100.0)	64 (100.0)	

* Chi-square test.

**Table 5:** Comparison of the two participant groups with respect to baseline health status.

	Variable	N (%)		*p* *
		Multidisciplinary	Multimodal	
Medication use	Yes	38 (59.4)	26 (40.6)	0.002
	No	17 (26.6)	11 (17.2)	
	As needed	9 (14.1)	27 (42.2)	
On sick leave	Yes	39 (60.9)	44 (68.8)	0.35
	No	25 (39.1)	19 (29.7)	
Physical exercise	Does not exercise	24 (37.5)	29 (45.3)	0.04
	Exercises once per week	9 (14.1)	11 (17.2)	
	Exercises twice per week	9 (14.1)	16 (25.0)	
	Exercises three times per week	6 (9.4)	4 (6.3)	
	Exercises four or more times per week	16 (25.0)	4 (6.3)	
Other chronic diseases	Yes	41 (64.1)	24 (37.5)	0.003
	No	23 (35.9)	40 (62.5)	
Comorbidity	No	23 (35.9)	39 (60.9)	0.01
	One	30 (46.9)	16 (25.0)	
	Two and more	11 (17.2)	9 (14.1)	
In total		64 (100.0)	64 (100.0)	

* Chi-square test.

**Table 6:** Comparison of the two participant groups with respect to baseline measurements of pain; health-related quality of life; degree of disability; depression, anxiety, and stress; and sleep quality.

		Median (25–75 %)		*p* *
		Multidisciplinary	Multimodal	
NRS		7.0 (6.0–8.0)	7.0 (6.0–8.0)	0.71
SF-36	Physical functioning	30.0 (17.5–50.0)	45.0 (25.0–65.0)	0.001
	Physical limitations	0.0 (0.0–0.0) (0–50.0) **	0.0 (0.0–0.0) (0–100.0) **	0.005
	Emotional limitations	0.0 (0.0–33.3)	16.5 (0.0–83.5)	0.04
	Energy and vitality	35.0 (22.5–50.0)	35.0 (25.0–45.0)	0.66
	Psychological health	56.0 (40.0–64.0)	52.0 (40.0–64.0)	0.89
	Social functioning	38.0 (25.0–50.0)	38.0 (38.0–50.0)	0.41
	Physical Pain	23.0 (23.0–33.0)	33.0 (23.0–45.0)	0.02
	General health	35.0 (25.0–47.5)	45.0 (30.0–55.0)	0.007
	Change compared with the previous year	12.5 (0.0–50.0)	25.0 (25.0–50.0)	0.01
ODI		41.0 (30.0–52.0)	34.0 (26.0–43.0)	0.004
DASS-21	Depression	5.0 (2.5–9.0)	4.0 (1.0–7.0)	0.09
	Anxiety	5.0 (2.0–8.0)	3.5 (1.0–6.0)	0.04
	Stress	7.0 (6.0–10.5)	7.0 (5.0–11.0)	0.42
PSQI	Sleep duration	2.0 (1.0–3.0)	1.0 (0.5–2.0)	0.01
	Sleep disturbances	2.0 (2.0–2.0)	2.0 (1.0–2.0)	0.10
	Sleep onset latency	2.0 (1.0–3.0)	2.0 (1.0–2.5)	0.56
	Difficulties due to insufficient sleep	1.0 (1.0–2.0)	1.0 (1.0–2.0)	0.51
	Sleep efficiency	2.0 (0.0–3.0)	1.0 (0.0–2.0)	0.14
	Sleep quality	2.0 (1.0–2.0)	1.5 (1.0–2.0)	0.10
	Use of pharmacological drugs	1.5 (0.0–3.0)	0.0 (0.0–2.5)	0.17
	In total	12.0 (8.0–15.0)	10.0 (6.0–13.0)	0.02

* Mann–Whitney U test. ** Total range.

**Table 7:** Comparison of baseline measurements of pain intensity; health-related quality of life; degree of disability; depression, anxiety, and stress; and sleep quality with measurements taken at the end of the multidisciplinary biopsychosocial program.

		Median (25–75 %)		*p* *
		Baseline	Post-Treatment	
NRS		7.0 (6.0–8.0)	5.0 (4.0–6.0)	<0.001
SF-36	Physical functioning	30.0 (17.5–50.0)	40.0 (30.0–55.0)	<0.001
	Physical limitations	0.0 (0.0–0.0)	0.0 (0.0–25.0)	<0.001
	Emotional limitations	0.0 (0.0–33.3)	33.3 (0.0–100.0)	<0.001
	Energy and vitality	35.0 (22.5–50.0)	50.0 (35.0–60.0)	<0.001
	Psychological health	56.0 (40.0–64.0)	66.0 (52.0–76.0)	<0.001
	Social functioning	38.0 (25.0–50.0)	50.0 (50.0–75.0)	<0.001
	Physical Pain	23.0 (23.0–33.0)	45.0 (35.0–45.0)	<0.001
	General health	35.0 (25.0–47.5)	45.0 (35.0–55.0)	<0.001
	Change compared with the previous year	12.5 (0.0–50.0)	75.0 (25.0–75.0)	<0.001
ODI		41.0 (30.0–52.0)	34.8 (28.4–43.1)	<0.001
DASS-21	Depression	5.0 (2.5–9.0)	3.0 (1.0–6.5)	<0.001
	Anxiety	5.0 (2.0–8.0)	3.0 (1.0–5.0)	<0.001
	Stress	7.0 (6.0–10.5)	5.5 (3.0–7.5)	<0.001
PSQI	Sleep duration	2.0 (1.0–3.0)	1.0 (0.0–2.0)	<0.001
	Sleep disturbances	2.0 (2.0–2.0)	2.0 (1.0–2.0)	0.001
	Sleep onset latency	2.0 (1.0–3.0)	2.0 (1.0–2.0)	0.005
	Difficulties due to insufficient sleep	1.0 (1.0–2.0)	1.0 (1.0–1.0)	0.06
	Sleep efficiency	2.0 (0.0–3.0)	0.0 (0.0–1.5)	<0.001
	Sleep quality	2.0 (1.0–2.0)	1.0 (1.0–2.0)	<0.001
	Use of pharmacological drugs	1.5 (0.0–3.0)	0.0 (0.0–2.0)	0.002
	In total	12.0 (8.0–15.0)	9.0 (5.5–1.0)	<0.001

* Wilcoxon test.

**Table 8:** Comparison of baseline measurements of pain intensity; health-related quality of life; degree of disability; depression, anxiety, and stress; and sleep quality with measurements taken at the end of multimodal treatment.

		Median (25–75 %)		*p* *
		Baseline	Post-Treatment	
NRS		7.0 (6.0–8.0)	4.0 (3.0–6.0)	<0.001
SF-36	Physical functioning	45.0 (25.0–65.0)	55.0 (32.5–65.0)	<0.001
	Physical limitations	0.0 (0.0–0.0)	0.0 (0.0–50.0)	0.02
	Emotional limitations	16.5 (0.0–83.5)	50 (0.0–100.0)	0.03
	Energy and vitality	35.0 (25.0–45.0)	45.0 (35.0–55.0)	<0.001
	Psychological health	52.0 (40.0–64.0)	62.0 (8.0–76.0)	<0.001
	Social functioning	38.0 (38.0–50.0)	50.0 (38.0–75.0)	<0.001
	Physical Pain	33.0 (23.0–45.0)	45.0 (32.5–55.0)	<0.001
	General health	45.0 (30.0–55.0)	45.0 (35.0–60.0)	0.01
	Change compared with the previous year	25.0 (25.0–50.0)	75.0 (25.0–75.0)	<0.001
ODI		34.0 (26.0–43.0)	30.0 (22.0–37.9)	<0.001
DASS-21	Depression	4.0 (1.0–7.0)	3.0 (1.5–7.0)	0.14
	Anxiety	3.5 (1.0–6.0)	3.0 (1.0–7.0)	0.51
	Stress	7.0 (5.0–11.0)	5.5 (3.0–9.0)	<0.001
PSQI	Sleep duration	1.0 (0.5–2.0)	1.0 (0.0–1.0)	0.001
	Sleep disturbances	2.0 (1.0–2.0)	2.0 (1.0–2.0)	0.07
	Sleep onset latency	2.0 (1.0–2.5)	1.0 (1.0–2.0)	<0.001
	Difficulties due to insufficient sleep	1.0 (1.0–2.0)	1.0 (1.0–2.0)	0.89
	Sleep efficiency	1.0 (0.0–2.0)	0.0 (0.0–1.0)	<0.001
	Sleep quality	1.5 (1.0–2.0)	1.0 (1.0–2.0)	0.003
	Use of pharmacological drugs	0.0 (0.0–2.5)	0.0 (0.0–2.0)	0.54
	In total	10.0 (6.0–13.0)	7.0 (5.0–10.5)	<0.001

* Wilcoxon test.

**Table 9:** Comparison of differences between baseline and post-treatment measurements between the two participant groups.

		Median (25–75 %)		*p* *
		Multidisciplinary	Multimodal	
NRS		−2.0 (−3.5, −1.0)	−3.0 (−4.0, −2.0)	0.33
SF-36	Physical functioning	10.0 (0.0–20.0)	5.0 (0.0–15.0)	0.05
	Physical limitations	0.0 (0.0–25.0)	0.0 (0.0–0.0)	0.36
	Emotional limitations	0.0 (0.0–33.3)	0.0 (0.0–33.3)	0.08
	Energy and vitality	10.0 (5.0–25.0)	5.0 (0.0–15.0)	0.04
	Psychological health	12.0 (4.0–20.0)	8.0 (0.0–16.0)	0.17
	Social functioning	12.5 (0.0–25.0)	12.5 (0.0–25.0)	0.21
	Physical Pain	12.5 (6.3–22.5)	10.0 (0.0–22.5)	0.09
	General health	10.0 (−5.0–15.0)	5.0 (−2.5–10.0)	0.14
	Change compared with the previous year	25.0 (0.0–50.0)	25.0 (0.0–50.0)	0.86
ODI		−4.2 (−12.7–2.0)	−4.0 (−8.0–0.0)	0.65
DASS-21	Depression	−2.0 (−5.0–0.0)	0.0 (−2.0–1.0)	<0.001
	Anxiety	−1.5 (−4.5–0.0)	0.0 (−1.0–1.0)	<0.001
	Stress	−2.0 (−5.0–0.0)	−1.5 (−3.5–0.0)	0.22
PSQI	Sleep duration	0.0 (−1.0–0.0)	0.0 (−1.0–0.0)	0.34
	Sleep disturbances	0.0 (−1.0–0.0)	0.0 (0.0–0.0)	0.32
	Sleep onset latency	0.0 (−1.0–0.0)	0.0 (−1.0–0.0)	0.82
	Difficulties due to insufficient sleep	0.0 (−1.0–0.0)	0.0 (0.0 - 0,0)	0.14
	Sleep efficiency	−1.0 (−1.0–0.0)	0.0 (−1.0–0.0)	0.35
	Sleep quality	0.0 (−1.0–0.0)	0.0 (−1.0–0.0)	0.64
	Use of pharmacological drugs	0.0 (−1.0–0.0)	0.0 (0.0–0.0)	0.04
	In total	−2.5 (−4.5, −0.5)	−2.0 (−4.0–0.5)	0.13

* Mann–Whitney U test.

**Table 10:** Differences in smartwatch-based measurements between the two participant groups.

	Median (25–75 %)		*p* *
	Multidisciplinary	Multimodal	
Sleep quality /score	76.8 (72.1–79.2)	77.3 (75.1–80.0)	0.12
Physical activity /step count	8.7071 (7.1420–10.6438)	9.1613 (8.0217–11.5786)	0.22

* Mann–Whitney U test.

**Table 11:** Association between sleep quality and physical activity assessed using the Fitbit smartwatch.

Pair of Variables		rho	95 % CI for rho	*p* *
Multidisciplinary biopsychosocial approach in the treatment of chronic low back pain				
Sleep quality	Physical activity	−0.234	−0.434, −0.013	0.04
Multimodal approach in the treatment of chronic low back pain				
Sleep quality	Physical activity	−0.199	−0.392–0.011	0.06

* Spearman correlation test.

## Data Availability

The data presented in this study are available upon request from the corresponding author.

## Supplementary Materials

The following supporting information can be downloaded at: https://www.mdpi.com/article/10.3390/ijerph23030350/s1, Table S1: Consort 2025 Expanded Checklist.

## Abbreviations

The following abbreviations are used in this manuscript:

WHO	World Health Organization
IASP	International Association for the Study of Pain
MBR	Multidisciplinary Biopsychosocial Rehabilitation
CLBP	Chronic Low Back Pain
EFIC	European Pain Federation
HRQoL	Health-Related Quality of Life
LBP	Low Back Pain
TENS	Transcutaneous electrical nerve stimulation
SF36	The 36-Item Short Form Health Survey
ODI	Oswestry Disability Index
ICC	Intraclass correlation coefficient
DASS-21	Depression, Anxiety and Stress Scale
NRS	Numeric rating scale
PSQI	Pittsburgh Sleep Quality Index
PMP	Pain Management Program
BMI	Body Mass Index
NICE	National Institute for Health and Care Excellence
PENS	Percutaneous Electrical Nerve Stimulation
CBT	Cognitive Behavioral Therapy
ACT	Acceptance and Commitment Therapy
QoLT	Quality of Life Technologies
